# Well-Annotated microRNAomes Do Not Evidence Pervasive miRNA Loss

**DOI:** 10.1093/gbe/evy096

**Published:** 2018-05-18

**Authors:** James E Tarver, Richard S Taylor, Mark N Puttick, Graeme T Lloyd, Walker Pett, Bastian Fromm, Bettina E Schirrmeister, Davide Pisani, Kevin J Peterson, Philip C J Donoghue

**Affiliations:** 1 School of Earth Sciences and School of Biological Sciences, University of Bristol, United Kingdom; 2 Department of Biology and Biochemistry, University of Bath, United Kingdom; 3 School of Earth and Environment, University of Leeds, United Kingdom; 4 Department of Ecology, Evolution and Organismal Biology, Iowa State University; 5 Department of Tumor Biology, Institute for Cancer Research, The Norwegian Radium Hospital, Oslo University Hospital, Norway; 6 Department of Biological Sciences, Dartmouth College, Hanover, New Hampshire

**Keywords:** microRNAs, annotation, evolution, birth, death, phylogeny

## Abstract

microRNAs are conserved noncoding regulatory factors implicated in diverse physiological and developmental processes in multicellular organisms, as causal macroevolutionary agents and for phylogeny inference. However, the conservation and phylogenetic utility of microRNAs has been questioned on evidence of pervasive loss. Here, we show that apparent widespread losses are, largely, an artefact of poorly sampled and annotated microRNAomes. Using a curated data set of animal microRNAomes, we reject the view that miRNA families are never lost, but they are rarely lost (92% are never lost). A small number of families account for a majority of losses (1.7% of families account for >45% losses), and losses are associated with lineages exhibiting phenotypic simplification. Phylogenetic analyses based on the presence/absence of microRNA families among animal lineages, and based on microRNA sequences among Osteichthyes, demonstrate the power of these small data sets in phylogenetic inference. Perceptions of widespread evolutionary loss of microRNA families are due to the uncritical use of public archives corrupted by spurious microRNA annotations, and failure to discriminate false absences that occur because of incomplete microRNAome annotation.

## Introduction

microRNAs (miRNAs) are short noncoding RNA molecules present in the genomes of animals, plants, fungi, and both green and brown algae (Tarver et al. 2012); they are important biomedically (Sayed and Abdellatif 2011), agriculturally (Zhou and Luo 2013), developmentally (Plasterk 2006), as causal agents of macroevolutionary change (Heimberg et al. 2008; Peterson et al. 2009), and in phylogenetic inference (Tarver et al. 2013). Though they are thought largely to evolve from random sequence, they have been perceived to be rarely lost after assuming a regulatory function (Tarver et al. 2013). Recent studies have called this prevailing view into question (Guerra-Assunção and Enright 2012; Meunier et al. 2013; Thomson et al. 2014; Hertel and Stadler 2015), arguing that miRNA families exhibit rates of loss approaching 80% in some species (Thomson et al. 2014; Hertel and Stadler 2015). However, there are two key challenges to inferring the evolutionary history of miRNAs. The first is incomplete annotation of the miRNAome of an organism as a consequence of incomplete genome and/or small RNA sequencing, an ascertainment bias that results in false-negatives (Thomson et al. 2014; Fromm et al. 2015). The second challenge is the misannotation of random or degraded sequence, or of other classes of small RNAs, as miRNAs (Chiang et al. 2010; Hansen et al. 2011; Wang and Liu 2011; Meng et al. 2012; Tarver et al. 2012; Castellano and Stebbing 2013; Taylor et al. 2014, 2017), which results in false-positives. The occurrence of false-positives is so widespread that some have argued that only 16% of metazoan miRNA annotations in the canonical miRNA database (Kozomara and Griffiths-Jones 2014) are genuine (Fromm et al. 2015).

In an attempt to obtain a realistic understanding of miRNA family evolution, we exploited a curated data set of eumetazoan miRNA families in which these biases are minimized (Fromm et al. 2015). This data set includes all metazoan taxa that have been the subject of exhaustive miRNAome annotation based on genome and small RNA transcriptome sequencing. We present an analysis of the dynamics of metazoan miRNA evolution, demonstrating that miRNA families are among the most conserved of phylogenetic characters. Most losses can be attributed to a small number of families that have been repeatedly lost, and a small number of lineages exhibiting reduced phenotypic complexity (e.g., as inferred from cell diversity; Valentine et al. 1994). Finally, our phylogenetic analyses of 1) the distribution of miRNA families, and 2) their nucleotide sequences, demonstrate the power of miRNAs in recovering phylogenetic resolution at the scale of the animal kingdom.

## Materials and Methods

### Taxon Sampling

To minimize false negatives we constrained our analyses to only those taxa with high-coverage genomic and small RNA sequencing. To eliminate the effect of transcriptional noise (i.e., non-miRNA sequences), we used the curated data set of Fromm et al. (2015) which is based on a reanalysis of the entire compliment of eumetazoan miRNA families in miRBase V.21 (Kozomara and Griffiths-Jones 2014) following established criteria (Ambros 2003; Kozomara and Griffiths-Jones 2011). Fromm and colleagues found that of the 7,095 annotated metazoan miRNA families in miRBase V.21, only 1,178 (16.60%) meet all of the criteria required for correct annotation, with a further 2,104 (29.7%) meeting some but not all of the criteria required for validation (e.g., lacking the products of both the 5′ and 3′ arms). Taxa with low coverage (<6×) genomes (e.g., *Taeniopygia guttata*), poorly annotated small RNA repertoires (e.g., *Ovis aries*), and uncertain phylogenetic position (e.g., *Strongyloides ratti*), were removed, reducing the data set to 1,139 miRNA families from 35 species, representing a broad range of metazoan taxa (supplementary file 1, Supplementary Material online). This data set minimizes both false negatives and any bias from misannotated miRNAs, providing the basis for an approximately unbiased assessment of the rates of miRNA family birth and death.

To establish the impact of data curation, we compiled an uncurated data set (supplementary file 2, Supplementary Material online) from miRBase v.21 (Kozomara and Griffiths-Jones 2014) which we subjected to parallel analyses. Not all of the taxa in our curated data set appear in miRBase v.21 (Kozomara and Griffiths-Jones 2014), viz. *Alligator mississippiensis*, *Melibe maugeana*, *Columba livia*, and *Chrysemys picta*, and so we included the curated annotations for these taxa in the otherwise uncurated data set. This facilitates direct comparison of the results of the analyses of the two data sets, but it biases the analysis in favor of the uncurated data set by raising the overall quality of annotation, minimizing the impact of noise reduction in the curated data set. Not all miRBase miRNA genes are assigned to families,—particularly in cases where there is only a single gene in the family. We included these as individual families containing the single gene. Some genes are named correctly but not assigned to the correct family. For example, in *Schmidtea mediterranea*, there are three genes which have correctly been given the name miR-7 but which are not included in the miR-7 family grouping on miRBase v.21 (Kozomara and Griffiths-Jones 2014). In such instances, we included each of these as a separate family. The alternative would have been to hand curate the data set, which would be unrepresentative of miRBase, or else we could assign genes to families based on their name. However, this would introduce additional artifact since many genes correctly assigned to families in miRBase have different names (e.g., mmu-mir-300 is currently correctly listed in the miR-154 family, not the miR-300 family, despite the difference in names). Thus, for consistency, we have followed the family groupings that are listed on miRBase and ignored the names of the genes.

### Phylogeny and Rates of Evolution

The phylogenetic relationships between taxa included in the analysis are not controversial and a constraint timetree was used based on previous molecular clock studies (Dabert et al. 2010; Pyron 2010; Erwin et al. 2011; Reis et al. 2011; Crête-Lafrenière et al. 2012; Tarver et al. 2016) onto which the evolution of microRNAs was mapped. This allowed us to estimate rates of miRNA gain and loss in two ways. First, for the analyses of the *Homo* and *Drosophila* lineages, numbers of shared miRNA families were calculated for the internal nodes of the phylogeny, so that the branching pattern could be observed. Second, for the total tree, the rate for each branch was averaged across the length of the branch with rates calculated as birth/death per million years. About 1,000 simulations were run to generate confidence intervals around the average values.

We investigated the phylogenetic and temporal distribution of gains and losses using a novel stochastic character mapping approach under a Dollo assumption; this model is employed even by those who argue that miRNA loss is prevalent (Thomson et al. 2014) since there is no evidence for the convergent evolution of miRNA families (Tarver et al. 2013). First, we assumed that characters were gained only once and that the gain occurred on either the terminal branch (for singletons), or the branch subtending the least inclusive clade of taxa exhibiting presence of that miRNA. Without further information to constrain its position, we assumed equal probability of miRNA family gain having occurred at any point along the branch and the precise timing of gain time was generated by randomly sampling from a uniform distribution between the beginning and the end of this branch. Thus, in practice, gains will always occur on the same branch, but the precise position (and hence timing) is determined stochastically and will vary each time the algorithm is run. Second, we assumed no losses occurred in the case of singletons but allowed stochastic losses to occur in instances of nonsingletons. Where losses were possible, these were simulated by using the make.simmap function in the phytools package (Revell 2012) by placing a hard prior probability of presence on the root, and then only allowing losses (1–0 transitions) in the model. When losses were observed, the branch on which they were inferred to have occurred was recorded, as well as the position along that branch (effectively a temporal estimate). This whole process was achieved using the custom-written function DolloSCM in the package Claddis (Lloyd 2016). Because the algorithm is stochastic, it was run 1,000 times in order to generate mean and 95% confidence intervals. Similarly, the output allows both phylogenetic and temporal positions to be examined for each inferred transition, which can themselves be subdivided into gains (0–1 transitions) and losses (1–0 transitions). We used this output to estimate per-branch rates for gains-only (as losses are necessarily biased both phylogenetically, toward nested branches, and temporally, toward the present, because they must occur after a gain). We also generated separate (gains and losses) time series of rates (changes per 10-Myr time bin) from 720 Ma to the present. Finally, we compared inferred losses across all miRNAs to simple minimum and maximum possible values based on the observed data. Here, the minimum was set at zero (no losses). The maximum is more complex to estimate, but here we used a simple rule, viz.: 1) if a singleton then no losses could be observed; 2) if presence of the miRNA is present in all members of a clade of two tips then, again, no losses could be observed; 3) if the least inclusive clade exhibiting the miRNA contains more than two taxa, a loss could be observed and the maximum number of losses is 2−*N*, where *N* is the number of taxa in that clade (i.e., with all losses occurring on terminal branches). These rules were then used to sum the maximum number of losses across all miRNAs. The inferred total number of losses can vary (due to the stochastic nature of the algorithm) and, thus, we considered the full distribution of our 1,000 runs when comparing between minimum and maximum losses. The Dollo model necessitates that characters are rarely lost and this may bias toward higher rates of gain. To avoid this potential artefact, we used a model that would produce much lower rates of gain compared with loss. Using DiscML (Kim and Hao 2014) in R, we estimated single rates of gain (0.16) and loss (1.64) for all characters, with the root set to miRNA absence. Using these estimates, we calculated maximum likelihood ancestral states in APE (Paradis et al. 2004) which we used to estimate rates of evolution in Claddis (Lloyd 2016). All analyses were performed in R (R Core Team 2016) and the code and data used are available from Dryad.

### Phylogenetics

To evaluate the suitability of miRNAs as phylogenetic characters, we first compiled a data matrix using the complete data set of all miRNA families, coding their presence and absence (supplementary file 1, Supplementary Material online). We then estimated the level of homoplasy in this data set using the Consistency Index, following Sanderson and Donoghue (1989, 1996), with comparisons made to their data set of 101 morphological and molecular matrices. The Consistency Index was calculated in MacClade with all characters considered to be unordered.

We conducted phylogenetic analyses of the curated and uncurated data sets (supplementary files 1 and 2, Supplementary Material online) using a stochastic Dollo binary substitution model (Nicholls and Gray 2008) implemented in RevBayes (Hohna 2016), with four discrete gamma loss rate categories across sites, and the homogeneous origination rate λ integrated out of the likelihood equation (see Alekseyenko et al. 2008). We applied an ascertainment bias correction to account for unobserved miRNA families lost in all species (Felsenstein 1992). We conducted a second analysis with all singleton families removed, applying a correction to account for the absence of both singletons as well as families lost in all species (Alekseyenko et al. 2008; Nicholls and Gray 2008). The RevBayes script used for this analysis is provided in supplementary file 3, Supplementary Material online. Convergence was assessed using the tracecomp and bpcomp programs in the PhyloBayes package (Lartillot et al. 2013), ensuring a maxdiff statistic < 0.05, a minimum effsize > 100 and maximum rel_diff < 0.1 across all parameters. Finally, we conducted phylogenetic analyses of concatenated pri-miRNA sequences. This alignment (supplementary file 4, Supplementary Material online) was run under a GTR + G model in PhyloBayes.

## Results

### Birth and Death of microRNA Families

**Fig. 1.**
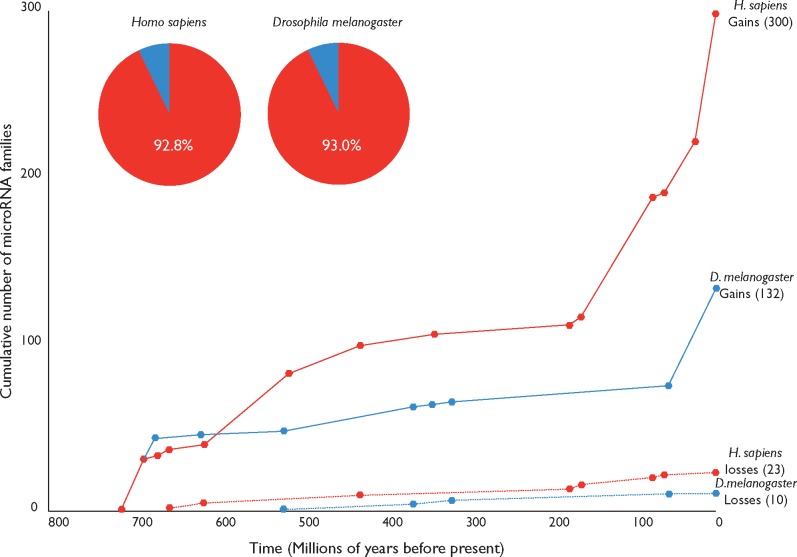
—Cumulative numbers of gains and losses of miRNA families in the lineages leading to the two most completely sampled taxa (*Homo* and *Drosophila)* over the past 720 Myr based on our curated data set.

The curated data set reveals a significant difference in the number of miRNA families present in the most completely annotated deuterostome (*Homo sapiens*) and protostome (*Drosophila melanogaster*), with 300 in *H. sapiens*, and 132 in *D. melanogaster* (fig. 1). This difference in cumulative miRNA family acquisition is widely recognized (Hertel 2006; Heimberg et al. 2008; Wheeler et al. 2009; Fromm et al. 2015); the large complement of human miRNAs inferred to be a consequence of episodic bursts in miRNA acquisition at the origin of bilaterians, vertebrates, placentals, and primates (Heimberg et al. 2008; Wheeler et al. 2009; Tanzer et al. 2010). Likewise, the absolute number of miRNA family losses varies between species (23 in *H. sapiens*; 10 in *D. melanogaster*). We find a low proportion of loss (∼0.07) in these lineages, with an overall ratio of 13.0 gains to every 1 loss in *H. sapiens* and 13.2 gains to each loss in *D. melanogaster*.

To determine the extent to which these lineages are representative of the broader pattern of miRNA gain and loss across metazoans, we mapped all character state changes onto the known phylogeny, with gains and losses calculated for each taxon, running from the root to tree tips. This reveals an average gain to loss ratio per taxon of 11.4 gains for every loss, equating to ∼0.08 loss proportion (table 1), with a median ratio of 10.5:1 and a loss proportion of ∼0.087.

**Table 1:** Sum Total of miRNA Family Gains and Losses Along the Path from the Root of the Phylogenetic Tree to Each Tip Based on our Curated Data Set

Node	Lineage	Gains	Losses	Gain:Loss Ratio	Percentage Loss
37	*Amphimedon*	8	0	N/A	N/A
38	*Nematostella*	28	0	N/A	N/A
40	*Capitella*	68	0	N/A	N/A
39	*Melibe*	61	2	30.50	3.28
47	*Apis*	77	4	19.25	5.19
46	*Ixodes*	57	3	19.00	5.26
54	*Branchiostoma*	56	3	18.67	5.36
52	*Saccoglossus*	54	3	18.00	5.56
56	*Petromyzon*	99	6	16.50	6.06
60	*Chrysemys*	130	8	16.25	6.15
63	*Columba*	154	10	15.40	6.49
48	*Tribolium*	88	6	14.67	6.82
61	*Alligator*	128	9	14.22	7.03
70	*Homo*	301	22	13.68	7.31
51	*D. melanogaster*	133	10	13.30	7.52
53	*Strongylocentrotus*	46	4	11.50	8.70
62	*Gallus*	146	13	11.23	8.90
71	*Macaca*	235	21	11.19	8.94
59	*Anolis*	124	12	10.33	9.68
67	*Bos*	219	22	9.95	10.05
50	*D. pseudoobscura*	99	10	9.90	10.10
44	*Celegans*	105	11	9.55	10.48
58	*Salmon*	113	12	9.42	10.62
57	*Danio*	115	13	8.85	11.30
49	*Bombyx*	88	10	8.80	11.36
66	*Sus*	198	24	8.25	12.12
69	*Mus*	259	33	7.85	12.74
65	*Monodelphis*	143	19	7.53	13.29
41	*Schmidtea*	58	9	6.44	15.52
64	*Ornithorhynchus*	129	21	6.14	16.28
68	*Rattus*	221	36	6.14	16.29
43	*Pristionchus*	140	25	5.60	17.86
42	*Ascaris*	64	14	4.57	21.88
45	*Tetranychus*	56	13	4.31	23.21
55	*Ciona*	40	14	2.86	35.00

Note.—The gain to loss ratio, and percentage loss, for each individual lineage are also shown. Thus, the three gains and two losses observed on the lineage leading to chordates are recorded in all descendent taxa.

**Fig. 2.**
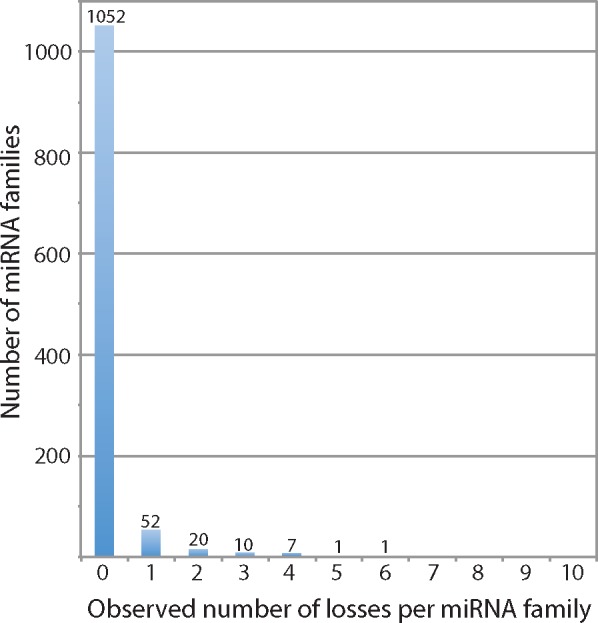
—The observed number of losses of miRNA families inferred from our phylogenetic analysis of the curated data set.

**Fig. 3.**
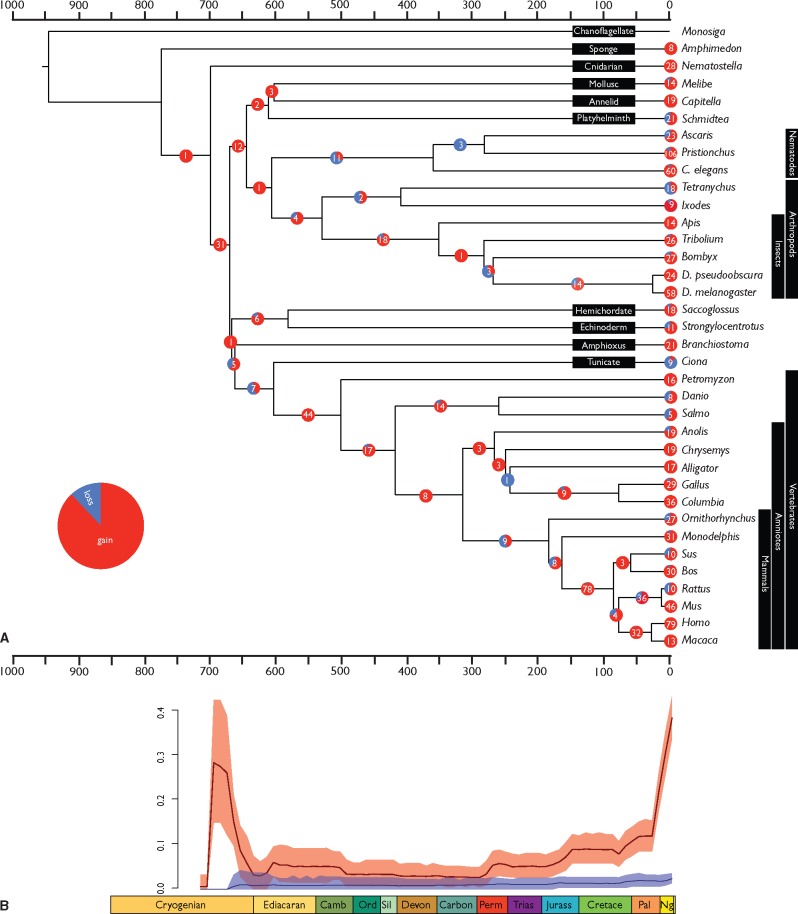
—The gains and losses of miRNA families for all taxa with well-annotated miRNAomes, (*A*) per branch within the phylogenetic tree, (*B*) per unit time across the phylogenetic tree. miRNA gains showed an increased rate toward the Recent, compatible with some theories which posit an early transient phase in which miRNA gain and loss is rapid (Peterson et al. 2009), making early losses difficult to detect.

Of the 1,143 miRNA families in the curated data set, 1,052 (92%) exhibit no losses, 52 are lost once, 20 are lost twice, 10 are lost three times, 7 are lost four times, miR-2001 is lost five times, and miR-315 is lost six times (fig. 2). Thus, although only 91 individual families exhibit losses, we observe a total of 161 losses, and these are distributed unevenly across the tree. Of the 68 branches with character changes, 24 show only gains of miRNA families, a further 20 show ≥ 75% gains, while only eight branches are characterized by more losses than gains (fig. 3*A*).

When character state changes are mapped through time (fig. 3*B*), average gains outweigh losses, although the confidence intervals overlap until the mid-Permian. Confidence intervals are constrained by relatively long branch lengths with an average length of 196 Myr, suggesting that the broad CIs reflect variable, rather than constant rates of character change. Thus, if we consider the 44 gains on the lineage leading to vertebrates (617–510 Ma), the CI spans a 6-fold range from 0.025 to 0.15 gains per million years. There is a significant increase in the evolution of miRNA families corresponding to the evolution of Bilateria during the Cryogenian. The rate decreases until an Ediacaran rise followed by a steady Cambrian–Permian decrease in the rate of gains. The subsequent rate of miRNA family gains has increased steadily, substantially from the Cretaceous. In contrast, losses exhibit an approximately constant (and low) rate from the Cryogenian. Numerous internal branches exhibit significantly high rates of miRNA family acquisition (e.g., stem-lineages of bilaterians, protostomes, vertebrates, placentals, and primates; supplementary file 5, Supplementary Material online). Some external branches show significantly high acquisition rates (most placentals, birds, drosophilids, and nematodes). Significantly low rates of miRNA family acquisition are observed on internal branches leading to mammals and birds. These results obtain regardless of whether the Dollo Parsimony or likelihood-based models of ancestral state reconstruction are used as a basis for analysis of the curated data set (supplementary file 6, Supplementary Material online). The likelihood-based analysis differs principally in inferring a greater flux of miRNA family gain and loss deep within metazoan evolution, though this is likely an analytic artefact of the uninformative outgroup that lacks miRNAs altogether (supplementary file 6, Supplementary Material online).

Analysis of the uncurated miRBase data set yielded results that imply an order of magnitude higher rate of gain and loss of miRNAs within the Cenozoic, toward the tips of the phylogenetic tree, in comparison to the rates inferred from the curated data set (supplementary file 7, Supplementary Material online).

### Utility of microRNA Families in Phylogenetics

The utility of a marker for phylogenetic inference is related to the level of homoplasy that it exhibits. In order to estimate the level of homoplasy in our miRNA family data set, we used the Consistency Index (CI) to characterize the number of times a miRNA family is gained and lost on the tree. CI is based on the minimum number of changes required by a character or data set, divided by the actual number of changes (Kluge and Farris 1969); values range from 0 to 1, where a CI of 1 reflects the minimum number of changes and, therefore, zero homoplasy. The expected CI can be inferred based on an empirically derived formula (Sanderson and Donoghue 1996); for a categorical data set of 35 taxa it is ∼ 0.50, and for molecular sequence data ∼ 0.64. In contrast, the CI observed for our miRNA data sets is 0.88, indicating that miRNA families scored simply by presence/absence, exhibit surprisingly low levels of homoplasy and, as such, should be reliable phylogenetic markers.

**Fig. 4.**
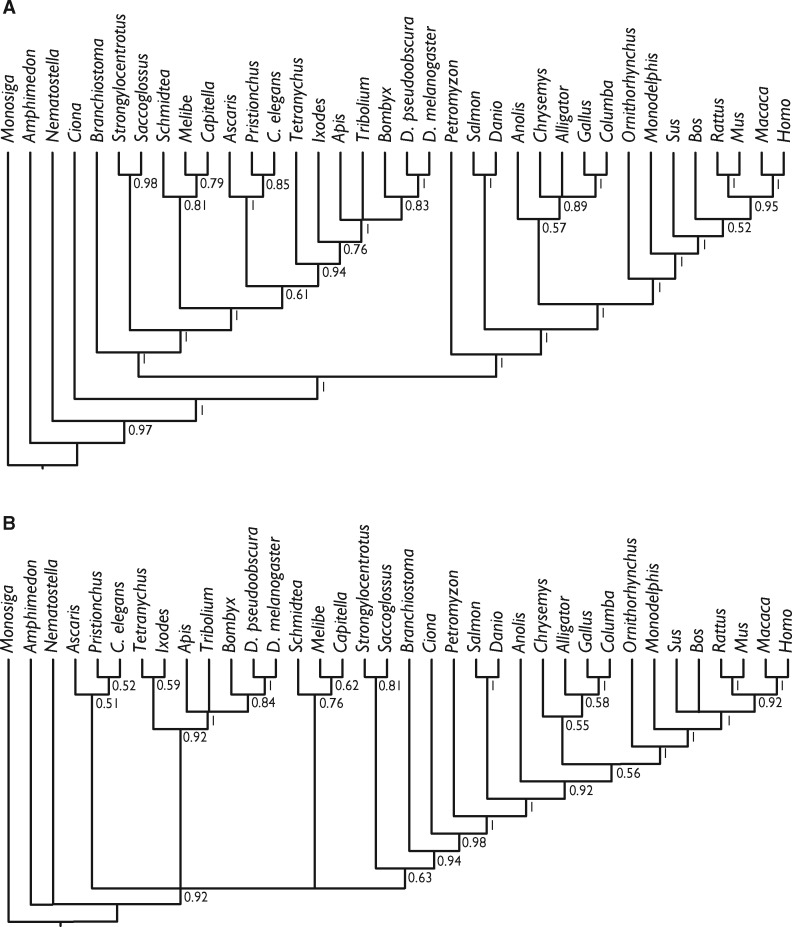
—Phylogenetic tree derived from the Dollo analysis of the curated presence/absence of microRNA families considered in our analysis. (*A*) Shows results whereby all miRNAs were included and (*B*) where singletons (synapomorphic) miRNAs were excluded. Node values are clade posterior probabilities.

**Fig. 5.**
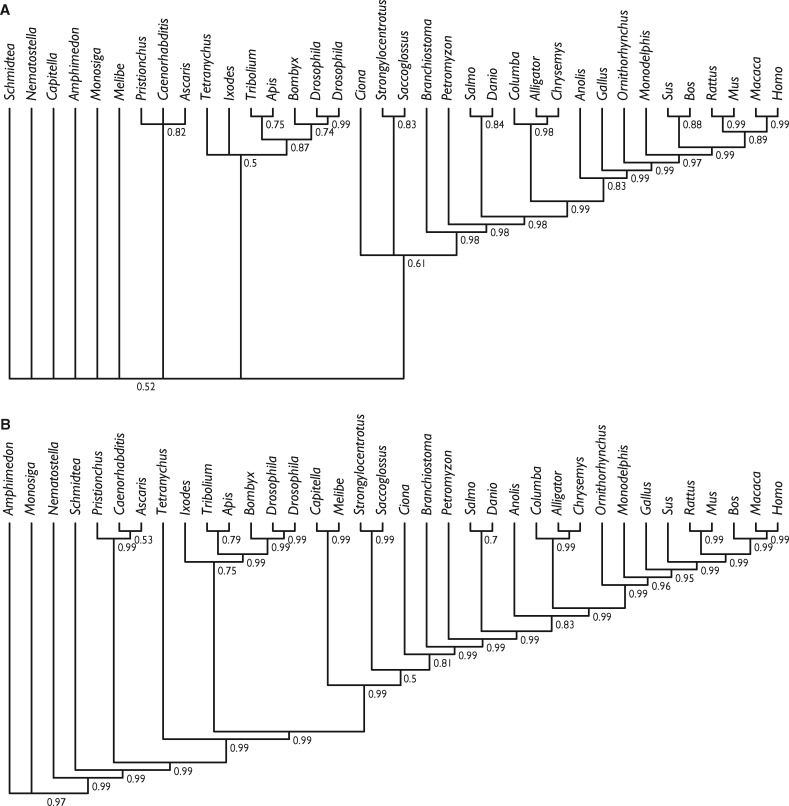
—Phylogenetic tree derived from the Dollo analysis of presence/absence of microRNA families in our uncurated miRBase data set. (*A*) Shows results whereby all miRNAs were included and (*B*) where singletons (synapomorphic) miRNAs were excluded. Node values are clade posterior probabilities.

Having established the empirical basis for the phylogenetic utility of miRNAs, we conducted a phylogenetic analysis of the same data set using a stochastic Dollo model of binary character evolution, implemented in RevBayes. This recovered trees that were largely compatible, even if not always highly supported, with current knowledge of metazoan evolution (fig. 4), suggesting that previous unorthodox results obtained using BEAST were at least partly driven by model mis-specification (previous analyses lacked ascertainment bias correction and did not use a Gamma distribution). Parallel analyses of the uncurated miRBase data set (supplementary file 2, Supplementary Material online) yielded trees that are much more incongruent with current perceptions of metazoan phylogeny (fig. 5), regardless of whether singletons were included (protostomes resolved as paraphyletic with respect to deuterostomes; lizards, and archosaurs resolved as paraphyletic with respect to mammals) or excluded (little resolution among invertebrates; lizard resolved as sister to mammals rather than archosaurs).

**Fig. 6.**
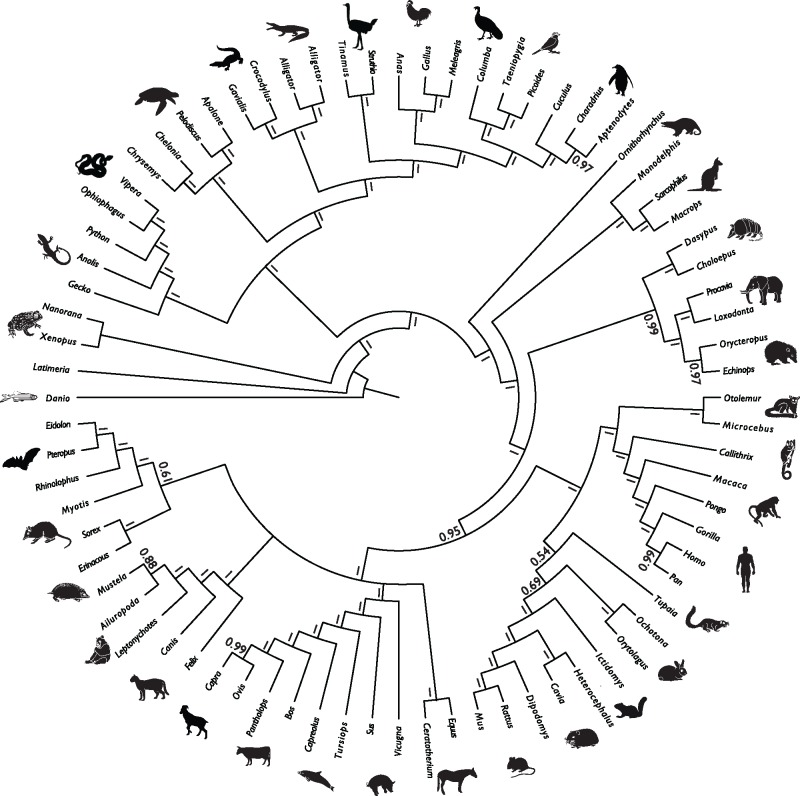
—Phylogenetic tree derived from phylogenetic analysis of the concatenated analysis of pri-miRNA sequences. Node values are clade posterior probabilities.

The results of the tetrapod superalignment are congruent with established phylogenies (fig. 6). Some parts of the tree, such as the higher level relationships within Laurasiatheria, are unresolved or have low levels of support, however, these nodes have traditionally proven hard to resolve (Tarver et al. 2016). This expanded data set provides additional support for turtles as archosaurs (Field et al. 2014), and resolves an Atlantogenata–Boreoeutheria root for placental mammals (Tarver et al. 2016).

## Discussion

### Diversification of miRNA Families

The results of our analyses based on the curated data set show highly variable rates in the diversification of miRNA families, with some internal branches showing significantly high acquisition rates (e.g., at the origins of Bilateria, Protostomia, Vertebrata, and Placentalia), while others show significantly low acquisition rates (e.g., at the origins of Aves, and Mammalia). Terminal branches also show highly variable rates; this likely reflects the depth of miRNA annotation rather than genuine biological signal, as many of these taxa are model organisms. The results of analyses of the uncurated miRBase data set imply several factors higher numbers and frequency of losses compared with the curated data set; the general pattern of miRNA family gain and loss is the same through the Phanerozoic, except for the Cenozoic where the uncurated data set implies an order of magnitude higher number and rate of gains and losses (supplementary file 7, Supplementary Material online). These differences reflect the impact of curation and the importance of using curated data from high coverage genomes in attempting to obtain an accurate perspective on miRNA family evolution; uncurated miRNA data sets yields spurious perceptions of miRNA evolution.

The diversification of microRNAs is the result of birth and death of individual miRNA families. Thus, apparent shifts in the miRNA diversification rate can be caused by 1) variable birth rates, 2) variable death rates, or 3) a combination of both. Previous work has suggested that shifts in miRNA diversification rates are caused by increased birth rates (Heimberg et al. 2008, 2010; Iwama et al. 2013; Platt et al. 2014), or constant birth rate and variable death rate (Lu et al. 2008; Nozawa et al. 2010; Quah et al. 2015). Our data support either variable birth rates, or a constant but low birth rate with slight variation in a low death rate, rather than high birth and death rates. High birth and death rates have been inferred primarily based on analyses of *Drosophila* species. Lu et al. (2008) has been criticized for misannotation of degraded transcriptional sequences as miRNAs (Berezikov 2010). Nozawa et al. (2010) also inferred high death rates of miRNA families in *Drosophila* but their study did not control for the validity of miRNAs in miRBase or variable genome quality, and compounded these problems by assaying miRNA evolution at the gene level, increasing false-negatives. Lyu et al. (2014) inferred 87–94% of miRNA family loss within 4–30 and 60 Myr of *Drosophila* evolution, respectively; unfortunately, their analysis assumed a constant birth rate a priori, for which there is no evidence.

Investigating butterflies and moths, Quah et al. (2015) observed that the terminal lineages in their phylogeny exhibited a much higher abundance of lineage-specific miRNAs than subtending lineages, supporting the view that miRNA family diversification rates are high. However, they failed to account for the antiquity of their terminal lineages (e.g., the species with the largest number of lineage-specific miRNAs, *Pararge aegeria*, is also estimated to have diverged from its sister taxa ∼100 Ma; Pohl et al. 2009; Wahlberg et al. 2009). It is this viewpoint, that lineage specific miRNAs are therefore young, which has been incorrectly interpreted as providing support for the high birth rate of miRNAs (Taylor et al. 2014). This artefact of perception underpins the theory of a transient phase of early miRNA evolution which now lacks an evidential basis. miRNAome annotation of populations and closely related species are required to assess the veracity of a perceived “pull of the Recent” in miRNA family acquisition rates.

### Loss of miRNAs

Overall, we observed low levels of miRNA family loss and the losses that we did observe are nonuniformly distributed across the phylogeny. The losses we observed provide support for the role of miRNA families in the evolution of morphological complexity (Sempere et al. 2006; Heimberg et al. 2008; Peterson et al. 2009; Wheeler et al. 2009; Berezikov 2011), since loss-prevalent lineages, such as *Ciona* (89%), *Ascaris* (22%), *Pristionchus* (14%), and *Schmidtea* (43%), correlate with phenotypic simplification (Erwin et al. 2011; Philippe et al. 2011; Fromm et al. 2013; Bai 2014), *contra*Dunn et al. (2014). Large proportions of losses are also observed in the spider mite *Tetranychus* (61%), which has undergone large scale genomic reduction, losing >27% of its protein coding genes (Grbic et al. 2011). Apparent exceptions to this are the relatively large proportions of miRNA families lost in *Ornithorhynchus* (30%) and *Rattus* (40%), and clustered losses within the murid lineage where 36% of the changes are losses. This branch shows the highest rate of loss (0.2 miRNA families per million years) in our data set. The miRNA losses identified in murids appear to be genuine, as evidenced by the comprehensively annotated genomes, deep coverage of its small RNA expression, which surpasses that of all other taxa, and also by synteny maps which showed that these losses of miRNA-families occurred on eight distinct chromosomes (Fromm et al. 2015). The nature of these losses is unexpected as nine of the eleven miRNAs that were lost, originated on the lineage leading to placentals. miRNA losses are otherwise mosaic in nature (Tarver et al. 2013), but the pattern here implies the loss of one or more closely associated genetic pathways, mirroring losses observed in the homeobox gene repertoire of murids (Zhong and Holland 2011).

### Comparisons to Studies Showing High Rates of Loss

While our results demonstrate that, overall, miRNA families exhibit proportionally few losses, and the majority of these are attributable to a small number of miRNAs families and evolutionary lineages, this pattern is inconsistent with the view that loss of miRNA families is close to zero (Sempere et al. 2006, 2007; Peterson et al. 2009; Sperling and Peterson 2009; Sperling et al. 2009; Wheeler et al. 2009; Tarver et al. 2013). Nevertheless, our results are clearly incongruent with those published in other studies suggesting high rates of microRNA family loss (Guerra-Assunção and Enright 2012; Meunier et al. 2013; Thomson et al. 2014; Hertel and Stadler 2015). All of these studies rely on different data sets, whether genomic (Guerra-Assunção and Enright 2012; Hertel and Stadler 2015), or small RNA (Meunier et al. 2013). Thus, the causes of the discrepancies between these studies and our own may include: 1) transcriptional noise, 2) false-negatives, and 3) false-positives.

#### Transcriptional Noise

The critical first step in understanding microRNA evolution is to establish a clear framework for what is and what is not a microRNA, that is to separate miRNAs (signal) from tRNAs, siRNAs, rRNAs, and degraded mRNAs (noise) within the transcriptome. The inclusion of degraded mRNAs, as well as other RNA molecules in studies of miRNA evolution, is a significant source of bias in the inference of miRNA losses, and has impacted previous results in several ways. First, individual studies such as Meunier et al. (2013), who identified many putative novel miRNAs and who showed variable conservation rates, are suspect given that of the 333 purported miRNAs candidates only 49 (14%) achieve all of the criteria necessary for miRNA annotation (Fromm et al. 2015). Second, such errors are compounded by the inclusion of such sequences into miRBase (Kozomara and Griffiths-Jones 2014) (the official online depository of microRNAs). Indeed, the majority of sequences deposited in miRBase may not be genuine microRNAs (Hansen et al. 2011; Wang and Liu 2011; Meng et al. 2012; Brown et al. 2013; Taylor et al. 2014; Fromm et al. 2015), but are instead fragments of a great many different types of RNA. Thus, in bioinformatic analyses (Guerra-Assunção and Enright 2012; Hertel and Stadler 2015) in which the miRNA catalogue of miRBase (Kozomara and Griffiths-Jones 2014) is used as a reference BLAST database, many of the sequences under investigation are not miRNAs, compromising the results from such studies.

#### False-Negatives

When inferring loss of miRNAs, a critical issue is the depth of genome sequencing, a problem noted but not addressed by Guerra-Assunçæo and Enright (2012) and Hertel and Stadler (2015). Issues associated with the use of low-coverage genomes in comparative genomics have been discussed previously by Milinkovitch et al. (2010) who showed that they can generate artificially high numbers of gene losses. Tarver et al. (2013) showed large-scale differences in the number of missing miRNAs between three levels of sequencing coverage, with complete, high, and low coverage genomes missing on an average 2.6%, 3%, and 19.5% miRNA families, respectively. Low-coverage genomes were missing, on average, 6 times more miRNA families than high-coverage genomes, yet low-coverage genomes were included in both Guerra-Assunçæo and Enright (2012) and Hertel and Stadler (2015).

Sequencing depth not only affects bioinformatic studies but also those that rely only on small RNA sequencing data, such as Meunier et al. (2013). This study generated small RNA-seq data from five organs of single male representatives of six species, used to investigate the pattern of mammalian miRNA evolution, concluding that there were high levels of miRNA family turnover (Meunier et al. 2013). However, miRNAs are known to have restricted tissue-specific expression profiles (Lagos-Quintana et al. 2002; Aboobaker et al. 2005), and so miRNA families present in the genome may not be sequenced in a sample of only five organs. Meunier et al. (2013) inferred the loss of miR-1, miR-22, and miR-122 (among others) in humans, despite numerous other studies highlighting the key roles these miRNA families play in muscle development (Zhao et al. 2005), cancer (Song et al. 2013), and in cholesterol and lipid metabolism within the liver (Girard et al. 2008). It is clear that false-negatives caused either by the use of low coverage genomes, unrepresentative tissue sampling, or reliance on small RNA sequencing without reference to a genome, have all greatly increased the number of apparent losses of miRNA families.

#### False-Positives

Genuine miRNAs incorrectly homologized in distantly related clades result in an overestimation of miRNA loss from all intermediate lineages. For example, the identification of mir-7880 in the 13-lined ground squirrel (Hertel and Stadler 2015), a miRNA family previously identified only in nematode intestinal parasites, implies 51 instances of loss across all intermediate lineages in the tree topology of Hertel and Stadler (2015). However, this miRNA is not only absent from the ground squirrel genome, its annotation as a miRNA is spurious (the hairpin sequence lacks a two nucleotide offset between the annotated 5′ and 3′ products; Fromm et al. 2015). Thus, all of these hypothesized losses are artefacts of a single false-positive.

### microRNAs and Phylogenetics

Data from our analysis of consistency indices suggest that miRNA families have the potential to be informative phylogenetic markers, supporting their use in previous studies (Heimberg et al. 2008, 2010; Sperling et al. 2009; Campbell et al. 2011; Philippe et al. 2011; Rota-Stabelli et al. 2011; Helm et al. 2012; Fromm et al. 2013; Tarver et al. 2013; Field et al. 2014). However, the phylogenetic utility of miRNA families has been called into question by Thomson et al. (2014) who found that the results from a selection of previous analyses lack statistical robustness. Following Tarver et al. (2013), Thomson et al. (2014) used a Bayesian stochastic Dollo model (Alekseyenko et al. 2008) which better accommodates homoplasy than does parsimony. This led to the reanalysis of some of the older studies using new genomic and additional small RNA-seq data (Field et al. 2014), correcting previous errors. Inevitably, early studies (e.g., Sperling et al. 2009; Heimberg et al. 2010; Philippe et al. 2011; Helm et al. 2012; Lyson et al. 2012) fall short of current standards, based as they were on low coverage miRNAome sequencing, annotated in the absence of a reference genome, and performed before the Bayesian stochastic Dollo model was available. As such, they warrant further investigation following contemporary standards of miRNAome sequencing depth (100 Ms reads) from a breadth of tissues, organs, and developmental stages, as well as manual annotation of the miRNAome supported by a (≥ 6×) reference genome and a high-quality bioinformatics pipeline (Taylor et al. 2017).

Our principal analyses were based on a data set that minimizes false-positives and negatives (supplementary file 1, Supplementary Material online). Our results demonstrate that miRNA families have a consistency index far higher than would be expected for either molecular or morphological data sets of similar taxonomic scale and, thus, should make robust phylogenetic markers. This is reflected in the results of our phylogenetic analyses that, even when based on miRNA family presence/absence, produces a tree that is in good agreement with phylogenies based on more traditional markers (fig. 4). In comparison to the result of a similar analysis by Tarver et al. (2013), many of the nodes exhibit lower support values, principally because of differences in taxon sampling which excluded lineages exhibiting few losses and introduced lineages that exhibit many losses. Nevertheless, the results are considerably more precisely and accurately resolved than those based on an uncurated miRBase data set (fig. 5; supplementary file 2, Supplementary Material online).

We found that support for some clades changed after including singleton miRNA families (fig. 4*A*), with stronger support for the conventional grouping of birds with reptiles, as well as a monophyletic Ecdysozoa. However, this analysis also gave strong support for a highly unconventional paraphyletic arrangement within Deuterostomia. These incongruities suggest that small miRNA families contain some phylogenetic signal but may still be subject to potentially strong partial sampling bias in our data set. Regardless, this topology was still largely congruent with many established metazoan relationships, despite the fact that nonsingletons accounted for only 322 miRNA families. Continued development of binary substitution models accommodating additional types of rate heterogeneity, as well as partial sampling schemes, may mitigate the effect of loss, leading to greater accuracy in such data sets (Taylor et al. 2014; Thomson et al. 2014).

Finally, the concatenation of pri-miRNA sequences into superalignments (Tarver et al. 2013) represents a promising approach for future analyses. This approach has already resolved relationships among taxa as diverse as mammals, primates, reptiles, drosophilids, and nematodes (Field et al. 2014; Kenny et al. 2015; Tarver et al. 2016), as well as in our analysis of amniotes. In our pre-miR analysis, the results are robust although there are some nodes which conflict with other recent studies, the critical taxa (e.g., horse; Tarver et al. 2016) have invariably been identified as phylogenetically problematic based on conflict between previously published studies. At the same time, these data resolve some controversial relationships, such as the earliest diverging lineage of placental mammals.

## Conclusions

Our data suggest that the loss of miRNA families is far from pervasive, contrary to several recent studies, all of which are undermined by a variety of biases, most substantially ascertainment bias. Where losses occur, they are nonrandomly distributed, with only 1.7% of miRNA families accounting for >45% of losses, while the nematodes *Ascaris* and *Pristionchus* account for 20% of losses yet represent only 6% of branches, bearing out the suggestion that miRNA family gains and losses are associated with increasing and decreasing phenotypic complexity (e.g., as inferred from cell diversity; Valentine et al. 1994), respectively. We demonstrate that the presence/absence of miRNA families has a consistency index higher than expected for either morphological or molecular data of comparable taxon sampling. Our phylogenetic analyses were, in the main, congruent with established metazoan relationships. However, our results suggest that small miRNA families (singletons in particular) may still be subject to partial sampling bias in our data set. Our data support a pattern of miRNA diversification caused either by variable rates of miRNA birth, or constant but low rates of miRNA birth and a variable (but low) death rate, rather than high birth and death rates. However, our understanding of miRNA evolution is greatly constrained by the paucity of taxa with well-annotated miRNAomes, as most of the taxa included here are distantly related to each other with an average lineage-specific branch length of 312.6 Myr. The paucity of taxa with well-annotated miRNAomes runs the risk of telescoping past flux in speciation and extinction onto individual internal branches of a phylogenetic tree. In this way, the summed effects of random processes can produce nonrandom bursts in (miRNA) innovation. More studies are needed to investigate miRNA family evolution among congeneric species with comparisons between the evolution of individual miRNA genes and the evolution of miRNA families. 

## Supplementary Material

Supplementary data are available at *Genome Biology and Evolution* online.

## Supplementary Material

Supplementary Data
