# Supplementary material for: Well-Annotated microRNAomes Do Not Evidence Pervasive miRNA Loss
Source: Genome Biol Evol. 2018 May 18;10(6):1457–70. doi: 10.1093/gbe/evy096 (PMC6007596; doi:10.1093/gbe/evy096)
Supplement: Supplementary Data [file evy096_suppl.zip › ESM_06_miRNA_rates_curated_likelihood.pdf]

ESM7 Figure 1: Mean per-branch rates (all transitions pooled)

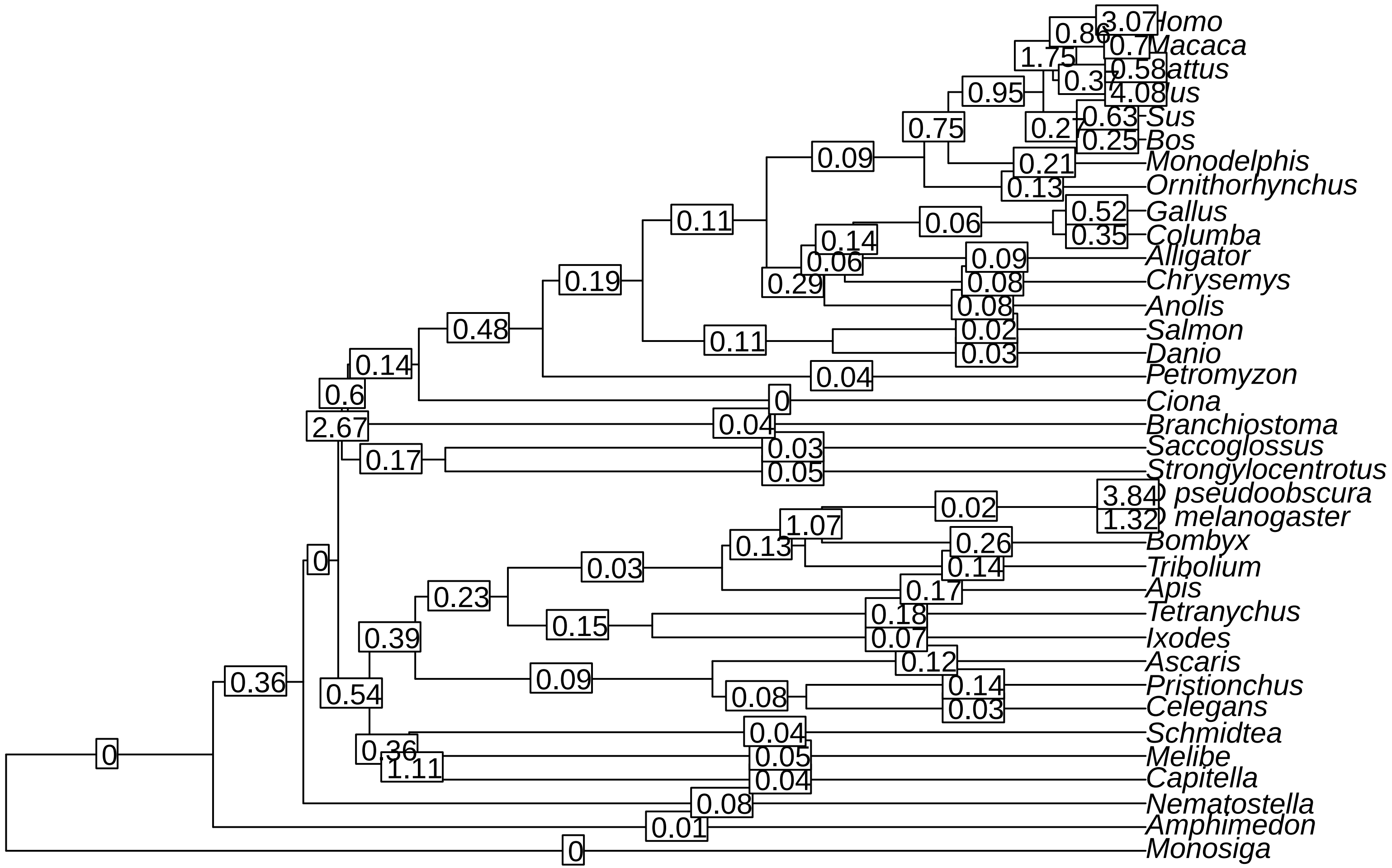

Electronic Supplementary Material 7: Results of the analysis of rates of miRNA evolution across Metazoa based on the curated dataset and likelihood-based analysis of ancestral states.

Electronic Supplementary Material 7: Results of the analysis of rates of miRNA evolution across Metazoa based on the curated dataset and likelihood-based analysis of ancestral states.

ESM7 Figure 2: Mean per-branch rates (gains only)

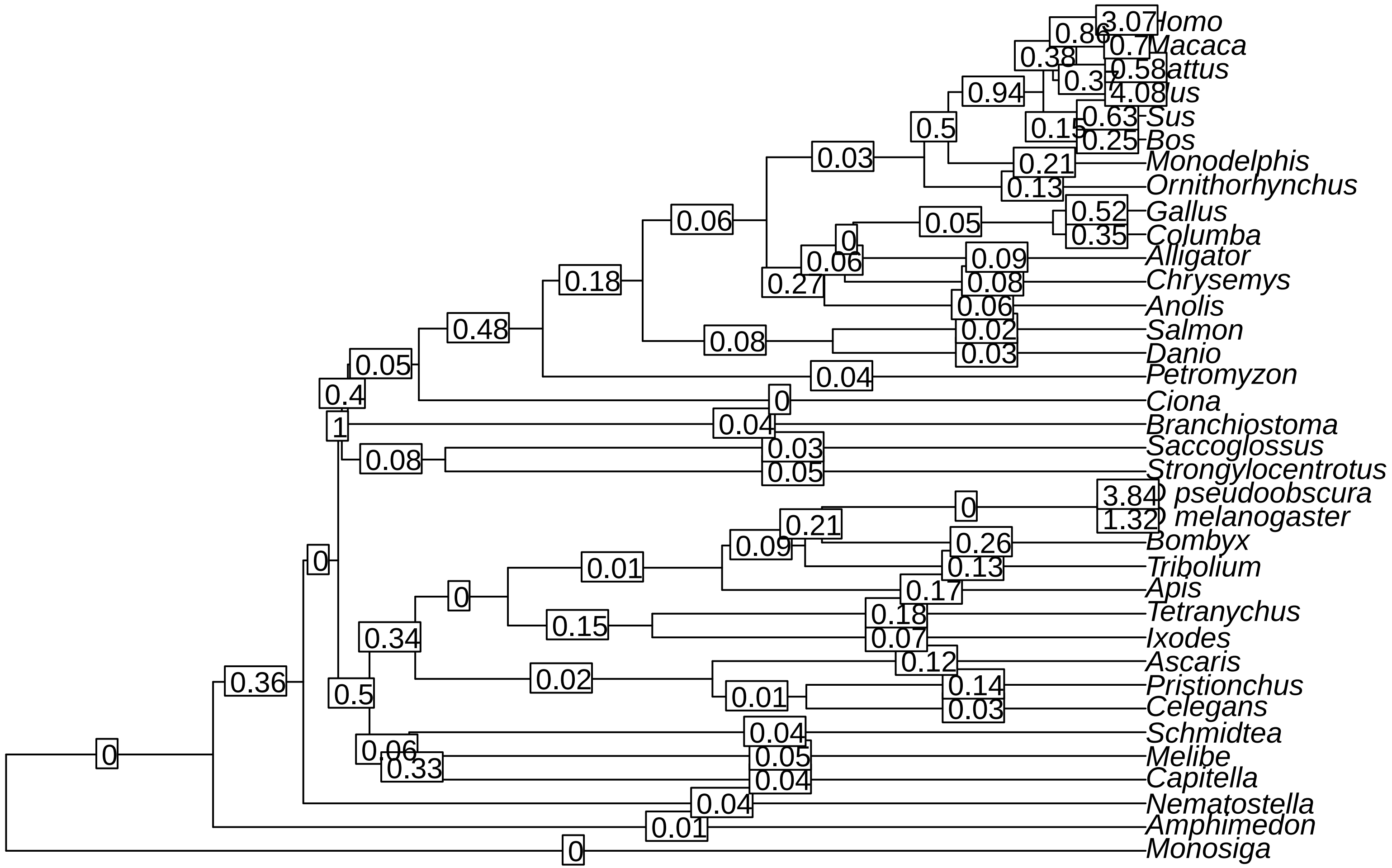

ESM7 Figure 3: Mean per−branch rates (gains only)
(Red = significantly high rate, Blue = significiantly low rate, White = non−significant rate)

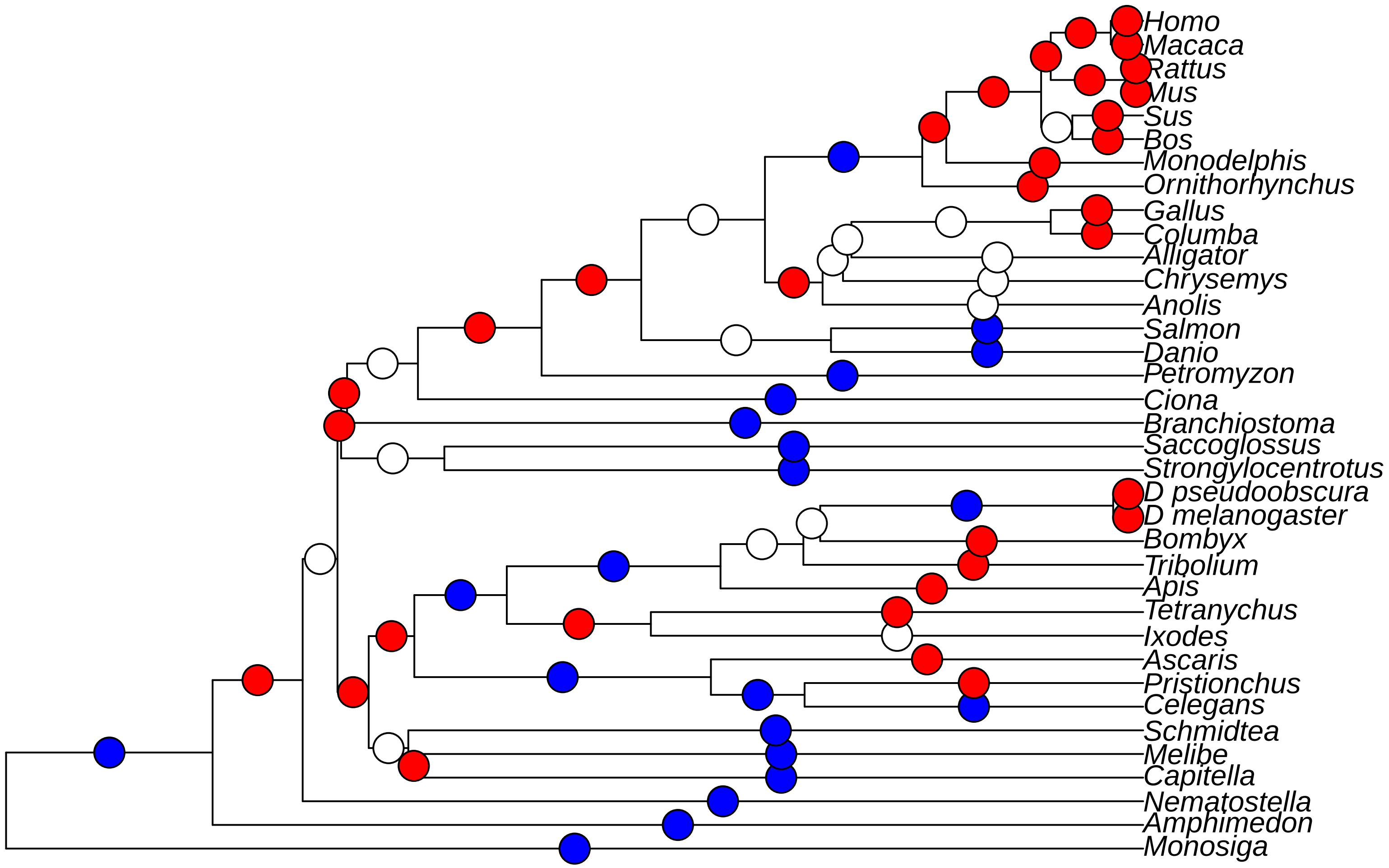

Electronic Supplementary Material 7: Results of the analysis of rates of miRNA evolution across Metazoa based on the curated dataset and likelihood-based analysis of ancestral states.

ESM7 Figure 4: Mean per−branch rates (losses only)

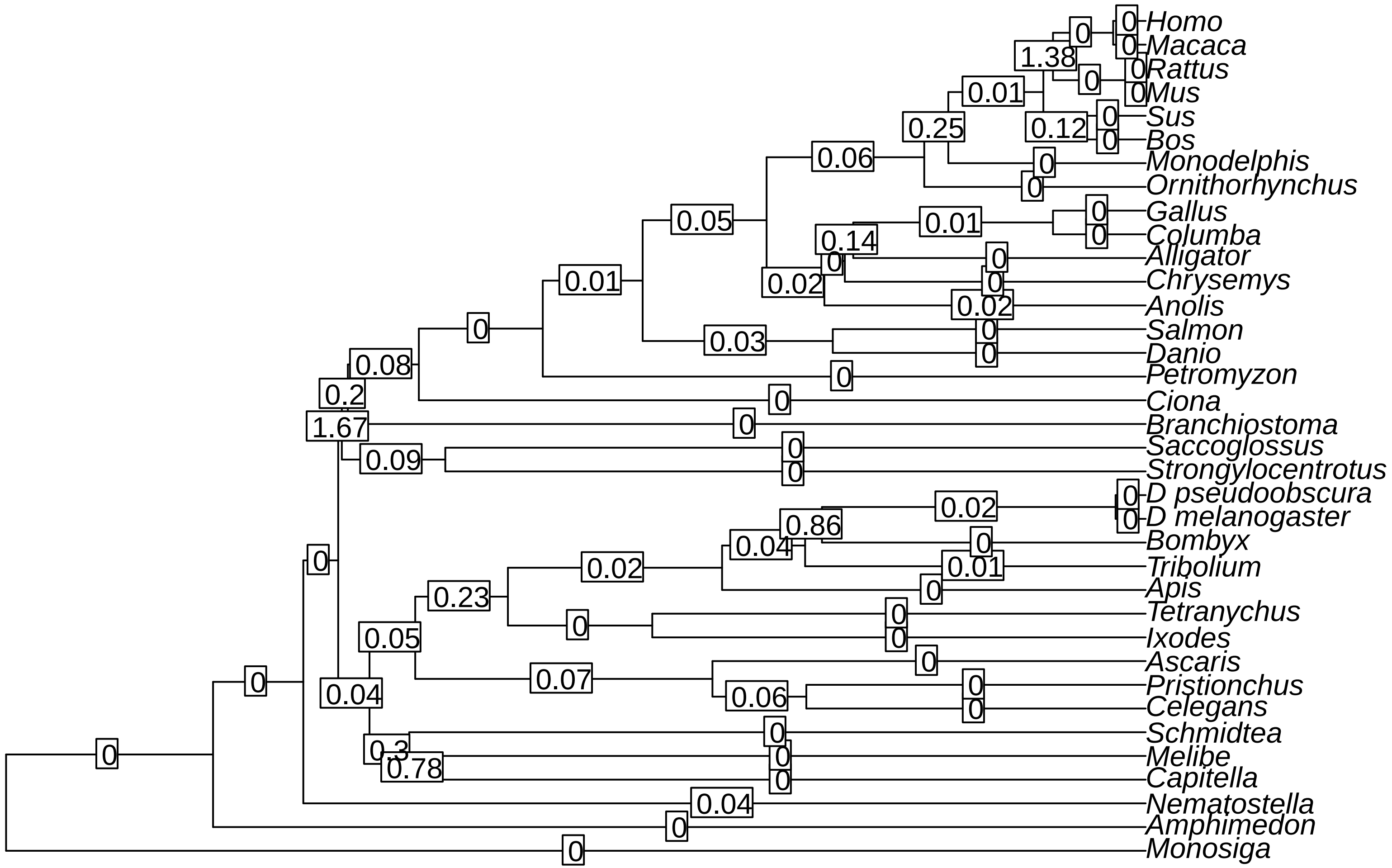

Electronic Supplementary Material 7: Results of the analysis of rates of miRNA evolution across Metazoa based on the curated dataset and likelihood-based analysis of ancestral states.

ESM7 Figure 5: Distribution of total losses across 1,000 runs of a stochastic algorithm. For comparison the minimum (zero) and maximum (2,585) possible number of losses are shown as vertical dotted lines. See ESM3 for how the latter value was calculated

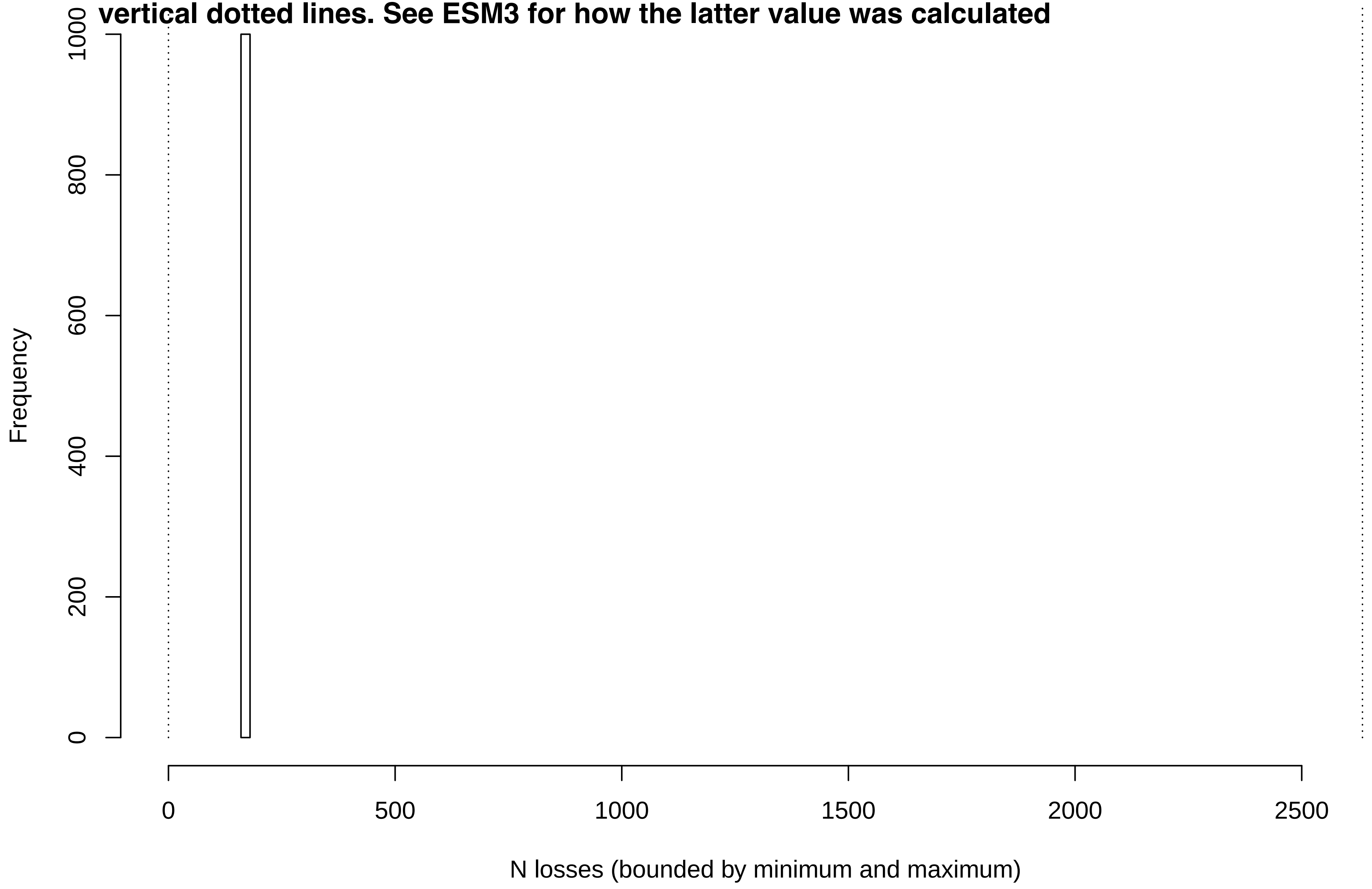

Electronic Supplementary Material 7: Results of the analysis of rates of miRNA evolution across Metazoa based on the curated dataset and likelihood-based analysis of ancestral states.

ESM7 Figure 6: Time series of per-lineage million year rates (all changes pooled) in 10-million-year bins (from 720 Ma to 0 Ma)

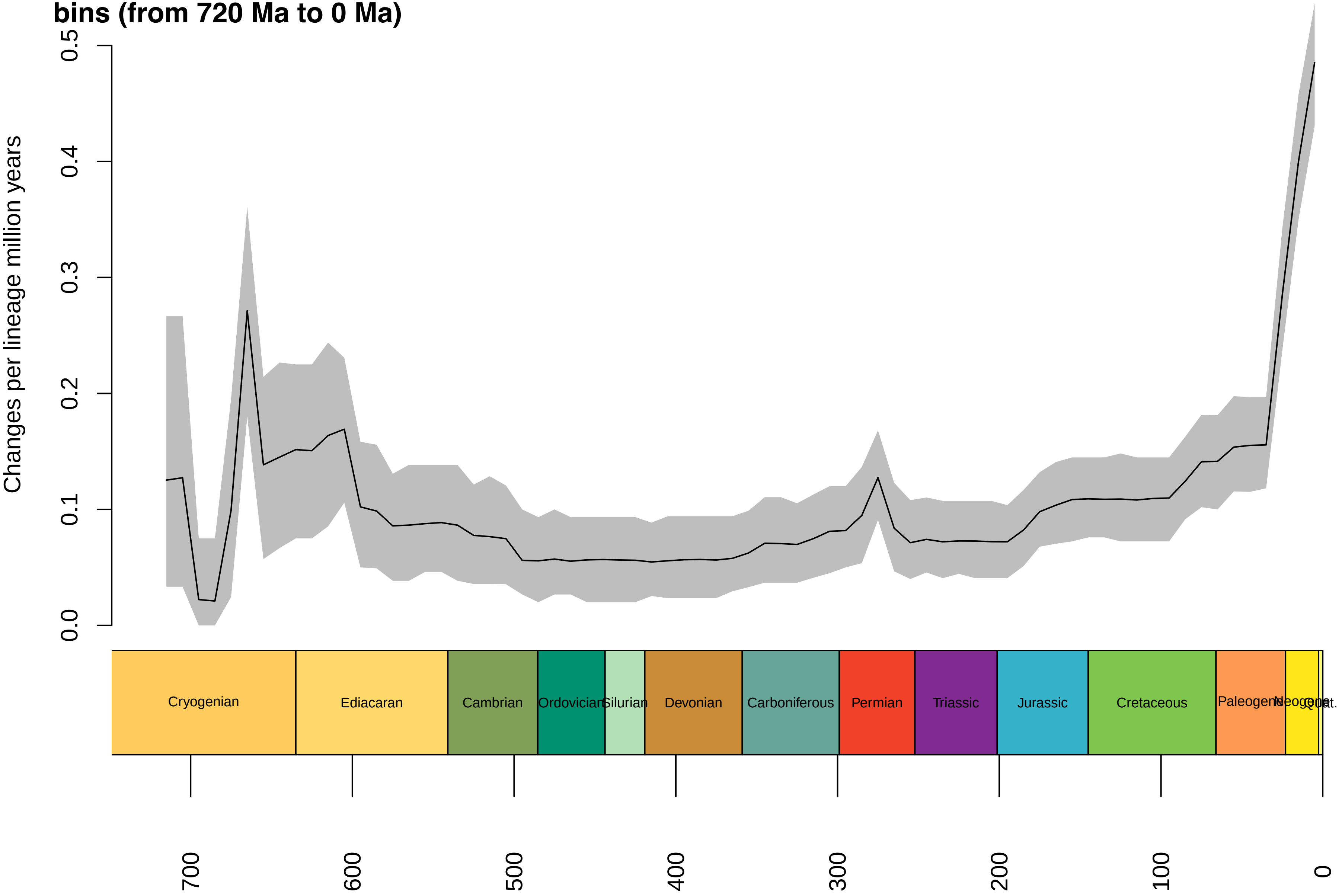

Electronic Supplementary Material 7: Results of the analysis of rates of miRNA evolution across Metazoa based on the curated dataset and likelihood-based analysis of ancestral states.

ESM7 Figure 7: Time series of per-lineage million year rates (gains only) in 10-million-year bins (from 720 Ma to 0 Ma)

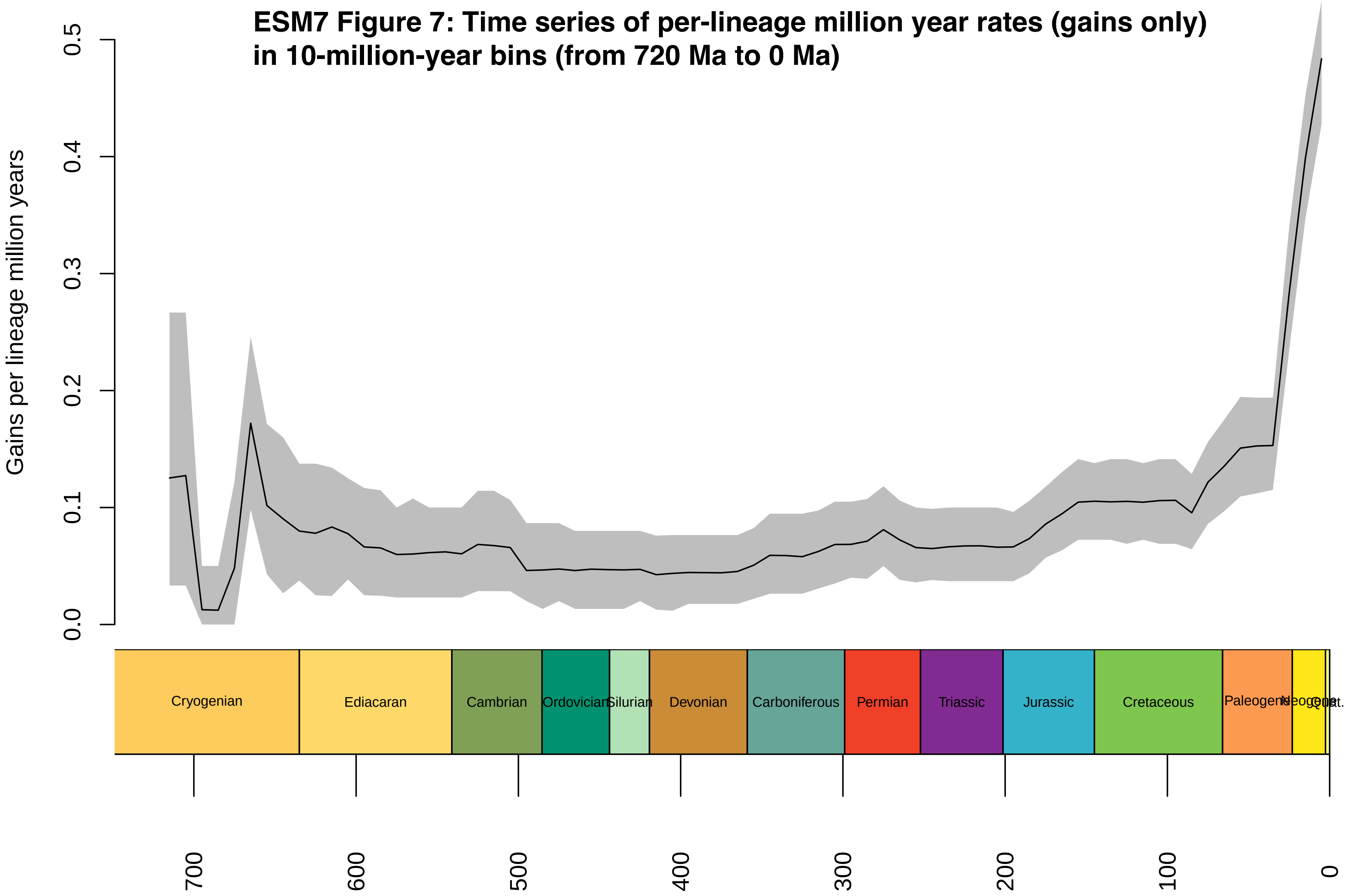

Electronic Supplementary Material 7: Results of the analysis of rates of miRNA evolution across Metazoa based on the curated dataset and likelihood-based analysis of ancestral states.

ESM7 Figure 8: Time series of per-lineage million year rates (losses only) in 10-million-year bins (from 720 Ma to 0 Ma)

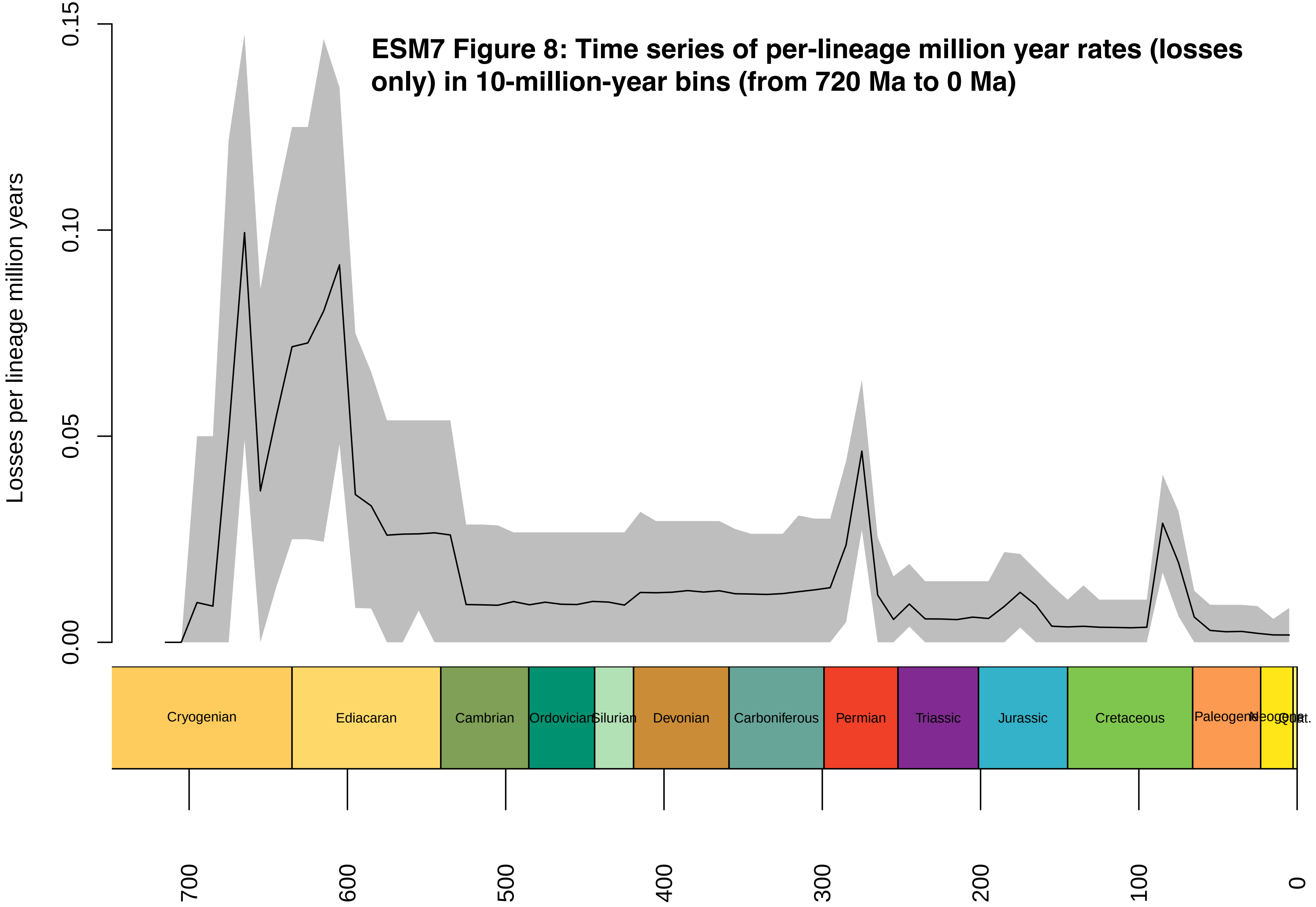

Electronic Supplementary Material 7: Results of the analysis of rates of miRNA evolution across Metazoa based on the curated dataset and likelihood-based analysis of ancestral states.

ESM7 Figure 9: Time series of per-lineage million year rates - Gains (red) and losses (blue) compared - in 10-million-year bins (from 720 Ma to 0 Ma)

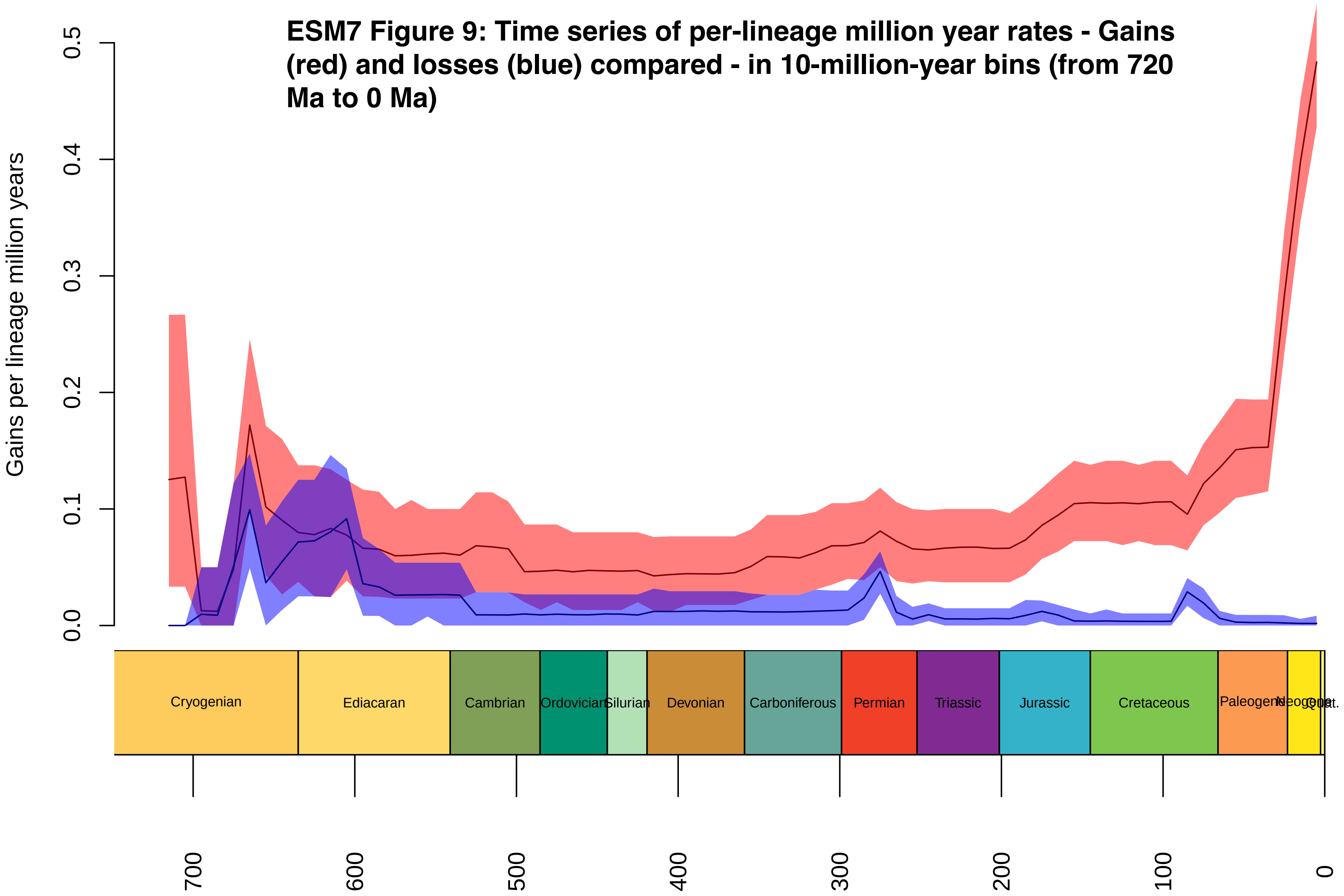

Electronic Supplementary Material 7: Results of the analysis of rates of miRNA evolution across Metazoa based on the curated dataset and likelihood-based analysis of ancestral states.
